# Transport of the multidrug resistance modulators verapamil and azidopine in wild type and daunorubicin resistant Ehrlich ascites tumour cells.

**DOI:** 10.1038/bjc.1990.225

**Published:** 1990-07

**Authors:** M. Sehested, T. Skovsgaard, P. B. Jensen, E. J. Demant, E. Friche, N. Bindslev

**Affiliations:** Dept of Pathology, Herlev University Hospital, Denmark.

## Abstract

**Images:**


Br. J. Cancer (1990), 62, 37-41 © Macmillan Press Ltd., 1990

### Transport of the multidrug resistance modulators verapamil and azidopine in wild type and daunorubicin resistant Ehrlich ascites tumour cells

M. Sehested[1], T. Skovsgaard[2], P. Buhl Jensen[3], E.J.F. Demant[5], E. Friche[4] & N. Bindslev[6]

[1]*Depts of Pathology and* [2]*Oncology, Herlev University Hospital, DK-2730 Herlev,* [3]*Depts of Oncology and* [4]*Internal Medicine, The Finsen Institute, DK-2100 Copenhagen and Depts of* [5]*Biochemistry C and* [6]*Biophysics and Physiology, The Panum Institute, DK-2200 Copenhagen N, Denmark.*

**Summary** Verapamil has been proposed to modulate the multidrug resistance phenotype by competitive inhibition of an energy dependent efflux of cytotoxic drug. However, the accumulation of both $^{14}$C-verapamil and $^{3}$H-verapamil was similar in wild type EHR2 and multidrug resistant EHR2/DNR+ Ehrlich ascites cells, and was much less in both cell lines in energy deprived medium than in medium containing glucose. Azidopine accumulation was also similar in both EHR2 and EHR2/DNR+ cells but, in contrast to verapamil, did not differ significantly with changes in cellular energy levels. Azidopine photolabelled a 170 kDa protein in EHR2/DHR+ plasma membrane vesicles which was immunoprecipitated by monoclonal antibody towards P-glycoprotein. Azidopine increased daunorubicin accumulation and modulated vincristine resistance in EHR2/DNR+ cells in a similar fashion to verapamil. Azidopine photolabelling was inhibited by vincristine and verapamil, but not by daunorubicin. Vincristine, but not daunorubicin, was able to increase both azidopine and verapamil accumulation in EHR2/DNR+ cells only. Finally, though both verapamil and azidopine are a substrate for P-glycoprotein in EHR2/DNR+ cells, they do not themselves appear to be transported by the multidrug resistance efflux mechanism to any significant extent in these cells.

The multidrug resistance (MDR) phenotype is characterised by: (1) cross-resistance between structurally and functionally unrelated drugs such as anthracyclines and vinca alkaloids; (2) decreased intracellular drug levels in resistant cells compared with wild type cells; (3) overexpression of a 170 kDa plasma membrane glycoprotein called P-glycoprotein; and (4) the ability of a number of amphipathic drugs to modulate resistance (recently reviewed by Bradley *et al.*, 1988). These modulators are considered to act by increasing cytotoxic drug levels in resistant cells by inhibiting their energy dependent efflux. It was therefore of interest to examine whether modulators had similar energy dependent accumulation patterns as the cytotoxic drugs in wild type and resistant cells, a finding which would be consistent with competitive inhibition for drug efflux. Verapamil, the most studied modulator which was first described by Tsuruo *et al.* (1981) and azidopine, a dihydropyridine analogue which has the advantage of being able to photoaffinity label P-glycoprotein (Safa *et al.*, 1987) were chosen for study.

### Materials and methods

### Cell lines

Wild type EHR2 and daunorubicin resistant EHR2/DNR + cells have previously been described in detail (Danø, 1971, 1973; Skovsgaard, 1978). EHR2/DNR + cells have all the characteristics associated with the multidrug resistance phenotype including cross resistance to vinca alkaloids and decreased drug accumulation (Skovsgaard, 1978), modulation by verapamil (Friche *et al.*, 1987), and increased expression of P-glycoprotein (Sehested *et al.*, 1989*a*).

### Chemicals

$^{3}$H-verapamil (81.1 Ci mmol$^{-1}$) was purchased from New England Nuclear (USA) while CN-$^{14}$-C-verapamil (12.9 mCi mmol$^{-1}$) was a generous gift from Knoll (FRG). $^{3}$H-azidopine (45.2 Ci mmol$^{-1}$) and unlabelled azidopine were from Amersham (UK). Nonidet P40 was from Shell (UK). ATP and vincristine were obtained from Sigma (USA) and daunorubicin from Farmitalia Carlo Erba (Italy). All other chemicals were of analytical grade.

### Measurement of drug accumulation in whole cells

Standard incubation medium was phosphate buffer pH 7.45 with 5% v/v dialysed calf serum and 10 mM glucose as previously described (Skovsgaard, 1977). Experiments with azidopine were performed in the dark. When depletion of cellular energy was required, glucose was omitted and 10 mM sodium azide added to the medium. After incubation with either $^{3}$H-azidopine, $^{3}$H-verapamil or $^{14}$C-verapamil cells were washed twice in ice-cold Ringer's solution by centrifugation, and the final pellet solubilised with 1 ml of 0.1% v/v Nonidet P40 overnight (solubilisation with either KOH or HCl yielded similar results). Control experiments with pelleting through silicone oil as described by Yusa *et al.* (1989) were also performed. Daunorubicin accumulation was measured by spectrofluorometry as previously described (Skovsgaard, 1977).

### Clonogenic assay

This was performed as previously described (Roed *et al.*, 1987). Only continuous incubation with drug for 3 weeks was used.

### Photoaffinity labelling with azidopine

Plasma membrane vesicles from EHR2 and EHR2/DNR + were photolabelled with $^{3}$H-azidopine as described by Safa *et al.* (1987). Photolabelled proteins were analysed by SDS-PAGE (9% gel), fluorography and photodensitometry. Immunoprecipitation of P-glycoprotein was performed as described by Mukhopadhyay and Kuo (1989) using the C219 monoclonal antibody against P-glycoprotein purchased from Centocor (Belgium).

### Results

### Accumulation of verapamil, azidopine and daunorubicin in whole cells

Figure 1 shows that the accumulation of $^{14}$C-verapamil in EHR2 and EHR2/DNR + is similar over the course of 2 h.

Correspondence: Maxwell Sehested, Dept. of Pathology, Rigshospitalet 5444, Blegdamsvej 9, DK-2100 Copenhagen, Denmark.
Received 27 November 1989; and in revised form 14 February 1990.

**Figure 1** Accumulation of $^{14}$C-verapamil in wild type EHR2 (**a**) and MDR EHR2/DNR + (**b**). Verapamil concentration was 5μM. Temperature 37°C. Bars equal s.e.m. of triple determinations. ▲, medium + 10 mM glucose; ■, medium + 10 mM glucose + 10 mM $NaN_3$; ●, medium + 10 mM $NaN_3$.

Furthermore, reducing the cellular energy generation by omission of glucose and addition of sodium azide demonstrates that deprivation of energy leads to very low levels of $^{14}$C-verapamil accumulation in both cell lines. This is further illustrated in Figure 2, where the addition of 10 mM glucose after 30 min of deprivation leads to a rapid increase in $^{14}$C-verapamil accumulation. Accumulation of $^{3}$H-verapamil (5 μM) at 60 min, 37°C was also equal in both cell lines and was likewise reduced by deprivation of cellular energy as accumulation of the $^{14}$C-isotope (not shown). The effect of daunorubicin, vincristine and azidopine on verapamil accumulation is shown in Figure 3. Daunorubicin inhibited verapamil accumulation in EHR2/DNR + by 15% at a 10-fold molar excess. The greater inhibition in EHR2 cells is presumably due to increased daunorubicin toxicity in the wild type cells. The decrease in verapamil accumulation in both EHR2 and EHR2/DNR + cells during simultaneous azidopine incubation can also be ascribed to azidopine toxicity at the 25 μM level. However, vincristine in 10–25-fold molar excess induced a small but statistically significant increase in verapamil accumulation in EHR2/DNR + cells only. Alteration of the cation composition in the incubation medium by exchanging $Na^+$ for $K^+$ up to 80 mM $K^+$ or by addition of $Ca^{2+}$ from 0 to 32 mM had no effect on verapamil accumulation (not shown). Neither did the use of a Tris instead of a phosphate buffer or when incubation media and pelleting and extraction procedures according to Broxterman et al. (1988), Kessel and Wilberding (1984) or Yusa et al. (1989) were used (not shown). Accumulation of verapamil was sensitive to variation in medium pH being approximately 50% higher at pH 7.45 than at pH 7.20 (not shown). Furthermore, verapamil accumulation was temperature dependent being approximately 50% lower at 4°C compared with 37°C (not shown), which is in agreement with findings by Kessel (1986).

**Figure 2** Accumulation of $^{14}$C-verapamil in EHR2/DNR + with 30 min incubation in medium containing 10 mM $NaN_3$ without glucose and then adding 10 mM glucose (●). Verapamil concentration was 5 μM. Temperature was 37°C. Accumulation in EHR2 followed the same pattern.

**Figure 3** Effect of azidopine, daunorubicin and vincristine on verapamil accumulation at 60 min, 37°C in EHR2 (**a**) and EHR2/DNR + (**b**). The histogram is composed of independent experiments with verapamil accumulation in standard medium containing 10 mM glucose as 100%. Bars equal s.e.m. of triple determinations. G, medium + 10 mM glucose with either 1, 2.5 or 5 μM $^{14}$C-verapamil or $^{3}$H-verapamil. $A_{10}$, medium + 10 mM glucose + 10-fold molar excess of azidopine (25 μM). $D_{10}$, medium + 10 mM glucose + 10-fold molar excess of daunorubicin (50 μM). $V_{10}$, medium + 10 mM glucose + 10-fold molar excess of vincristine (10μM). $V_{25}$, medium + 10 mM glucose + 25-fold molar excess of vincristine (25 μM). ●, $P < 0.02$ (Student's $t$ test) versus G.

On the other hand, the accumulation pattern of azidopine was entirely different from that of verapamil as demonstrated in Figure 4 in that accumulation is rapid and with only minor effect of manipulation of cellular energy levels. The decrease in azidopine levels over time in medium containing sodium azide in both cell lines suggest that this is due to an additive toxic effect. However, as for verapamil, steady state azidopine levels are similar in both cell lines. Azidopine increased daunorubicin accumulation in EHR2/DNR + cells in a dose dependent manner (Figure 5). Finally, though a 10-fold molar excess of daunorubicin had no effect on azidopine accumulation, there was a slight but statistically significant dose dependent effect of verapamil in 5–25-fold molar excess on azidopine accumulation in EHR2/DNR + cells but not in EHR2 cells (Figure 6). Vincristine in 10-fold molar excess also significantly increased azidopine accumulation in EHR2/DNR + cells (Figure 6).

**Figure 4** Accumulation of $^3$H-azidopine in EHR2 (**a**) and EHR2/DNR + (**b**) at 37°C. Azidopine concentration was 2 µM. ○, medium + 10 mM glucose; ●, medium + 10 mM $NaN_3$.

**Figure 5** Dose–response curve of the effect of increasing concentration of azidopine on daunorubicin accumulation in EHR2/DNR + cells in medium containing 10 mM glucose measured at 60 min, 37°C. Daunorubicin concentration in medium was 5 µM. $NaN_3$ line was daunorubicin accumulation in medium without glucose + 10 mM $NaN_3$.

**Figure 6** Influence of vincristine, verapamil and daunorubicin on $^3$H-azidopine accumulation in EHR2 (**a**) and EHR2/DNR + cells (**b**) measured at 60 min, 37°C. Azidopine concentration was 1 µM. Bars equal s.e.m. of triple determinations. A, medium + 10 mM $NaN_3$. G, medium + 10 mM glucose. $D_{10}$, medium + 10 mM glucose + 10 µM daunorubicin. $V_{10}$, medium + 10 mM glucose + 10 µM vincristine. $VP_5$, medium + 10mM glucose + 5 µM verapamil. $VP_{10}$, medium + 10 mM glucose + 10 µM verapamil. $VP_{25}$, medium + 10 mM glucose + 25 µM verapamil. ●, $P<0.05$ (Student's *t* test) versus G. ●●, $P<0.01$ versus G. ●●●, $P<0.05$ versus $VP_5$. ●●●●, $P<0.001$ versus $VP_{10}$.

**Figure 7** Photoaffinity labelling of plasma membrane vesicles with azidopine. Membrane vesicles from EHR2 and EHR2/DNR + were labelled with $^3$H-azidopine at 0.41 µM. Fluorographs were obtained after SDS-PAGE (50 µg protein per lane). Lane 1, EHR2. Lanes 2–6 EHR2/DNR +: lane 3, + 83 µM unlabelled azidopine; lane 4, + 42 µM verapamil; lane 5, + 42 µM vincristine; lane 6, + 42 µM daunorubicin. Lane 7, immunoprecipitation of $^3$H-azidopine labelled P-glycoprotein.

*Photoaffinity labelling and immunoprecipitation of P-glycoprotein*

$^3$H-azidopine photolabelled a 170 kDa protein in plasma membrane vesicles from EHR2/DNR + only (Figure 7, lanes 1 and 2), and immunoprecipitation with monoclonal antibody C219 confirmed azidopine labelling of P-glycoprotein (Figure 7, lane 7). In repeated experiments, vincristine inhibited labelling of P-glycoprotein as measured by densitometry scans by 41–80% at 100-fold molar excess (e.g. Figure 7, lane 5). Verapamil inhibited azidopine labelling by 38–73% at 100-fold molar excess (e.g. Figure 7, lane 4), while daunorubicin did not inhibit azidopine labelling in any of three experiments at 100-fold molar excess (e.g. Figure 7, lane 6).

*Clonogenic assay*

Both verapamil and azidopine act as typical multidrug resistance modifiers in EHR2/DNR + cells (Figure 8), azidopine being more efficient as modulator than verapamil but also more toxic by itself. Separate experiments showed that verapamil was more toxic to EHR2 cells with an $IC_{50}$ of 55 µM than to EHR2/DNR + with an $IC_{50}$ of 90 µM (not shown).

**Figure 8** Clonogenic assay demonstrating modulating ability of 1.0 μM (————) and 2.5 μM (- - - -) azidopine compared with 2.5 μM (......) and 5 μM (+ - + -) verapamil on vincristine resistance in EHR2/DNR + cells. (— — — —) is vincristine alone. Bars equal s.e.m. of triple determinations.

### Discussion

The MDR1 gene mRNA is overexpressed in a number of human tumours (Fojo *et al.*, 1987) and the modulation of the MDR phenotype could therefore well have clinical importance. The overexpression of P-glycoprotein appears to be essential to the MDR phenotype of increased drug efflux (Bradley *et al.*, 1988) and the relationship between modulator and P-glycoprotein has therefore received a great deal of attention. Verapamil is the best documented modulator since its description by Tsuruo *et al.* (1981), and has also been shown to inhibit daunorubicin efflux and modulate resistance in our EHR2/DNR + cell line (Friche *et al.*, 1987 and Figure 8). Verapamil has been reported to have an accumulation pattern typical of drugs in the MDR family, i.e. decreased accumulation in MDR cells compared to wild type cells in medium containing glucose (Broxterman *et al.*, 1988; Cano-Gauchi & Riordan, 1987; Kessel & Wilberding, 1984; Warr *et al.*, 1988; Yusa and Tsuruo, 1989) and increased accumulation in resistant cells after deprivation of cellular energy sources (Kessel & Wilberding, 1984). However, as demonstrated in Figures 1 and 2, the accumulation pattern of verapamil is similar in both EHR2 and EHR2/DNR + cells and furthermore shows the opposite reaction to deprivation of cellular energy to that of MDR drugs like daunorubicin and vincristine in the same cell lines (Skovsgaard, 1978). This difference in the verapamil accumulation pattern in EHR2 and EHR2/DNR + cells compared with other described MDR cell lines is hardly due to experimental procedures as we have tried a variety, including copies of those of Broxterman *et al.* (1988), Kessel and Wilberding (1984) and Yusa and Tsuruo (1989) and have furthermore also used two different verapamil isotopes. It is also unlikely that the lack of a demonstrable verapamil efflux 'pump' in EHR2/DNR + is due to its being overshadowed by a more rapid influx as influx is slower than for daunorubicin (Figure 1 and Skovsgaard, 1978). Interestingly, several MDR cell lines have been reported to be hypersensitive to verapamil itself (Cano-Gauchi & Riordan, 1987; Twentyman *et al.*, 1986; Warr *et al.*, 1988). This is not the case for EHR2/DNR + which has a higher $IC_{50}$ value of 90 μM verapamil compared to 55 μM for EHR2 in a 3-week continuous incubation clonogenic assay, a cross resistance which is clearly not caused by reduced accumulation (Figure 1). Recently other murine MDR cell lines which are also cross resistant to verapamil have been described (Reeve *et al.*, 1989).

The dihydropyridine analogue azidopine has received great interest in characterisation of the MDR phenotype since its original description by Safa *et al.* (1987) as it is inherently photoreactive (Bruggemann *et al.*, 1989; Kamiwatari *et al.*, 1989; Schurr *et al.*, 1989; Yang *et al.*, 1988; Yoshimura *et al.*, 1989). However, to our knowledge, the modulating properties of azidopine as well as its own cellular accumulation have not been previously described. As shown in Figures 5 and 8, azidopine is a typical modulator with an efficiency exceeding that of verapamil in EHR2/DNR + cells (Figure 5 and Friche *et al.*, 1987), though it is considerably more cytotoxic by itself than verapamil (Figure 8). It typically photolabels P-glycoprotein in plasma membrane vesicles from EHR2/DNR + cells (Figure 7) as previously described in other MDR cell lines (Safa *et al.*, 1987). Thus, though azidopine binds to P-glycoprotein, there is no difference in its accumulation between wild type and resistant cells (Figure 4). In fact, the accumulation of azidopine is remarkably similar to that reported for nitrendipine (Kessel, 1986). Similar results have been described for the modulating bisbenzylisoquinoline alkaloid cepharanthine, which is also accumulated in equal amounts in wild type and resistant cells (Shiraishi *et al.*, 1987) and which also inhibits azidopine labelling of P-glycoprotein (Asoh *et al.*, 1989; Kamiwatari *et al.*, 1989).

Yusa and Tsuruo (1989) reported that a 1,740-fold excess of vinblastine was able to increase $^3H$-verapamil accumulation by 40% in MDR K562/ADM cells thus reaching to a level of 43% of that of wild type K562 cells. We also found that vincristine significantly increased verapamil accumulation in EHR2/DNR + cells (Figure 3) and furthermore that vincristine and verapamil also increased azidopine accumulation (Figure 6) to levels which exceeded those found in wild type EHR2 cells. This curious phenomenon of drug levels in MDR cells exceeding those in wild type cells has not, to our knowledge, previously been described. It is not readily explained, but could be due to altered intracellular distribution of these modulating drugs caused by activation of P-glycoprotein.

Recently, two azidopine binding sites on P-glycoprotein have been detected (Bruggemann *et al.*, 1989), with the C terminal one being mostly labelled (Yoshimura *et al.*, 1989). Azidopine photolabelling of P-glycoprotein in EHR2/DNR + is inhibited by a large (100-fold) molar excess of both vincristine and verapamil to roughly the same extent as described by Safa *et al.* (1987), but not by daunorubicin (Figure 7). Safa also found only a slight (19%) inhibition of azidopine labelling by a 200-fold molar excess of another anthracycline, namely doxorubicin. This inhibition pattern of azidopine photolabelling is in agreement with results in Figure 6 where both vincristine and verapamil significantly increase azidopine accumulation in EHR2/DNR + but not EHR2 cells while daunorubicin has no effect. Furthermore, although verapamil at a 5-fold molar excess increases daunorubicin accumulation in EHR2/DNR + at least 400% (Friche *et al.*, 1987), daunorubicin does not increase verapamil accumulation even at 10-fold molar excess (Figure 3), a result which is in agreement with Kessel (1986). Vincristine however, whose accumulation in EHR2/DNR + cells is increased at least 500% by a 10-fold molar excess of verapamil (Sehested *et al.*, 1989*b*) is only able to increase verapamil accumulation by 37% at a similar molar excess (Figure 3). Thus, daunorubicin appears to interact differently with P-glycoprotein in EHR2/DNR + cells compared to azidopine, verapamil and vincristine.

In conclusion, though azidopine and verapamil are substrates for P-glycoprotein in EHR2/DNR + cells and could thereby modify P-glycoprotein function, they do not themselves follow the MDR efflux pathway to any detectable degree in these cells.

Supported by the Danish Cancer Society (grant 90-044). The expert technical assistance of Marianne Knudsen, Inge Kobbernagel, Annette Nielsen, Eva Høj and Vibeke Sejer is gratefully acknowledged.

### References

ASOH, K.-I., SABURI, Y., SATO, S.-I., NOGAE, I., KOHNO, K. & KUWANO, M. (1989). Potentiation of some anticancer agents by dipyrimadole against drug-sensitive and drug-resistant cancer cell lines. *Jpn. J. Cancer Res.*, **80**, 475.

BRADLEY, G., JURANKA, P.F. & LING, V. (1988). Mechanism of multiple drug resistance. *Biochem. Biophys. Acta*, **948**, 87.

BROXTERMAN, H.J., PINEDO, H.M., KUIPER, C.M., KAPTEIN, L.C.M., SCHUURHUIS, G.J. & LANKELMA, J. (1988). Induction by verapamil of a rapid increase in ATP consumption in multidrug-resistant tumor cells. *FASEB J.*, **2**, 2278.

BRUGGEMANN, E.P., GERMANN, U.A., GOTTESMAN, M.M. & PASTAN, I. (1989). Two different regions of phosphoglycoprotein are photoaffinity-labeled by azidopine. *J. Biol. Chem.*, **264**, 15483.

CANO-GAUCHI, D.F. & RIORDAN, J.R. (1987). Action of calcium antagonists on multidrug resistant cells. Specific cytotoxicity independent of increased cancer drug accumulation. *Biochem. Pharmacol.*, **36**, 2115.

DANØ, K. (1971). Development of resistance to daunomycin (NSC-82151) in Ehrlich ascites tumor. *Cancer Chemother. Rep.*, **55**, 133.

DANØ, K. (1973). Active outward transport of daunomycin in resistant Ehrlich ascites tumor cells. *Biochem. Biophys. Acta*, **323**, 466.

FOJO, A.T., UEDA, K., SLAMON, D.J., POPLACK, D.G., GOTTESMAN, M.M. & PASTAN, I. (1987). Expression of a multidrug-resistance gene in human tumors and tissues. *Proc. Natl Acad. Sci. USA*, **84**, 265.

FRICHE, E., SKOVSGAARD, T. & NISSEN, N. (1987). Effect of verapamil on daunorubicin accumulation in Ehrlich ascites tumor cells. *Cancer Chemother. Pharmacol.*, **19**, 35.

KAMIWATARI, M., NAGATA, Y., KIKUCHI, H. & 6 others (1989). Correlation between reversing of multidrug resistance and inhibiting of $^3$H azidopine photolabeling of P-glycoprotein by newly synthesized dihydropyridine analogues in a human cell line. *Cancer Res.*, **49**, 3190.

KESSEL, D., (1986). Interactions among membrane transport systems: anthracyclines, calcium antagonists and anti-estrogens. *Biochem. Pharmacol.*, **35**, 2825.

KESSEL, D. & WILBERDING, C. (1984). Mode of action of calcium antagonists which alter anthracycline resistance. *Biochem. Pharmacol.*, **33**, 1157.

MUKHOPADHYAY, T. & KUO, M.T. (1989). Expression of the P-glycoprotein gene in multidrug-resistant Chinese hamster ovary cells. *Anticancer Res.*, **9**, 575.

REEVE, J.G., WRIGHT, K.A., RABBITTS, P.H., TWENTYMAN, P.R. & KOCH, G. (1989). Collateral resistance to verapamil in multidrug-resistant mouse tumor cells. *J. Natl Cancer Inst.*, **81**, 1588.

ROED, H., CHRISTENSEN, I.J., VINDELØV, L.L., SPANG-THOMSEN, M. & HANSEN, H.H. (1987). Inter-experiment variation and dependence on culture conditions in assaying the chemosensitivity of human small cell lung cancer cell lines. *Eur. J. Cancer Clin. Oncol.*, **23**, 177.

SAFA, A.R., GLOVER, C.J., SEWELL, J.L., MEYERS, M.B., BIEDLER, J.L. & FELSTED, R.L. (1987). Identification of the multidrug resistance-related membrane glycoprotein as an acceptor for calcium channel blockers. *J. Biol. Chem.*, **262**, 7884.

SCHURR, E., RAYMOND, M., BELL, J.C. & GROS, P. (1989). Characterization of the multidrug resistance protein expressed in cell clones stably transfected with the mouse mdr1 cDNA. *Cancer Res.*, **49**, 2729.

SEHESTED, M., BINDSLEV, N., DEMANT, E.J.F., SKOVSGAARD, T., & JENSEN, P.B. (1989*a*). Daunorubicin and vincristine binding to plasma membrane vesicles from daunorubicin-resistant and wild type Ehrlich ascites tumor cells. *Biochem. Pharmacol.*, **38**, 3017.,

SEHESTED, M., JENSEN, P.B., SKOVSGAARD, T. & 4 others (1989*b*). Inhibition of vincristine binding to plasma membrane vesicles from daunorubicin-resistant Ehrlich ascites cells by multidrug resistance modulators. *Br. J. Cancer*, **60**, 809.

SHIRAISHI, N., AKIYAMA, S.-I., NAKAGAWA, M., KOBAYASHI, M. & KUWANO, M. (1987). Effect of bisbenzylisoquinolone (biscoclaurine) alkaloids on multidrug resistance in KB human cancer cells. *Cancer Res.*, **47**, 2413.

SKOVSGAARD, T. (1977). Transport and binding of daunorubicin, adriamycin, and rubidazone in Ehrlich ascites tumour cells. *Biochem. Pharmacol.*, **26**, 215.

SKOVSGAARD, T. (1978). Mechanism of cross-resistance between vincristine and daunorubicin in Ehrlich ascites tumor cells. *Cancer Res.*, **38**, 4722.

TSURUO, T., IIDA, H., TSUKAGOSHI, S. & SAKURAI, Y. (1981). Overcoming of vincristine resistance in P388 leukemia in vivo and in vitro through enhanced cytotoxicity of vincristine and vinblastine by verapamil. *Cancer Res.*, **41**, 1967.

TWENTYMAN, P.R., FOX, N.E., & BLEEHAN, N.M. (1986). Drug resistance in human lung cancer lines: cross-resistance studies and effects of the calcium transport blocker, verapamil. *Int. J. Radiat. Oncol. Biol. Phys.*, **12**, 1355.

WARR, J.R., ANDERSON, M. & FERGUSSON, J. (1988). Properties of verapamil-hypersensitive multidrug-resistant Chinese hamster ovary cells. *Cancer Res.*, **48**, 4477.

YANG, C.-P., H., MELLADO, W. & HORWITZ, S.B. (1988). Azidopine photoaffinity labeling of multidrug resistance-associated glycoproteins. *Biochem. Pharmacol.*, **37**, 1417.

YOSHIMURA, A., KUWAZURU, Y., SUMIZAWA, T. & 4 others (1989). Cytoplasmic orientation and two-domain structure of the multidrug transporter, P-glycoprotein, demonstrated with sequence-specific antibodies. *J. Biol. Chem.*, **264**, 16282.

YUSA, K. & TSURUO, T. (1989). Reversal mechanism of multidrug resistance by verapamil: direct binding of verapamil to P-glycoprotein on specific sites and transport of verapamil outward across the plasma membrane of K562/ADM cells. *Cancer Res.*, **49**, 5002.


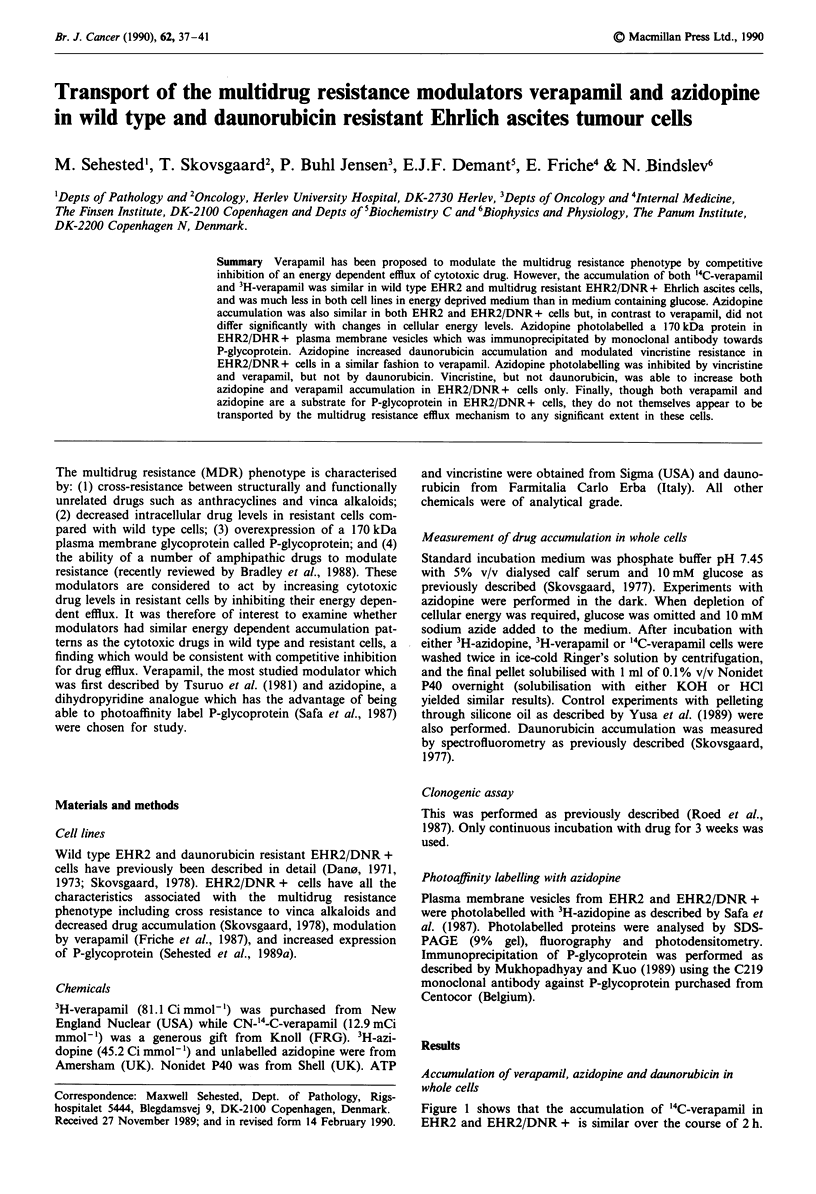

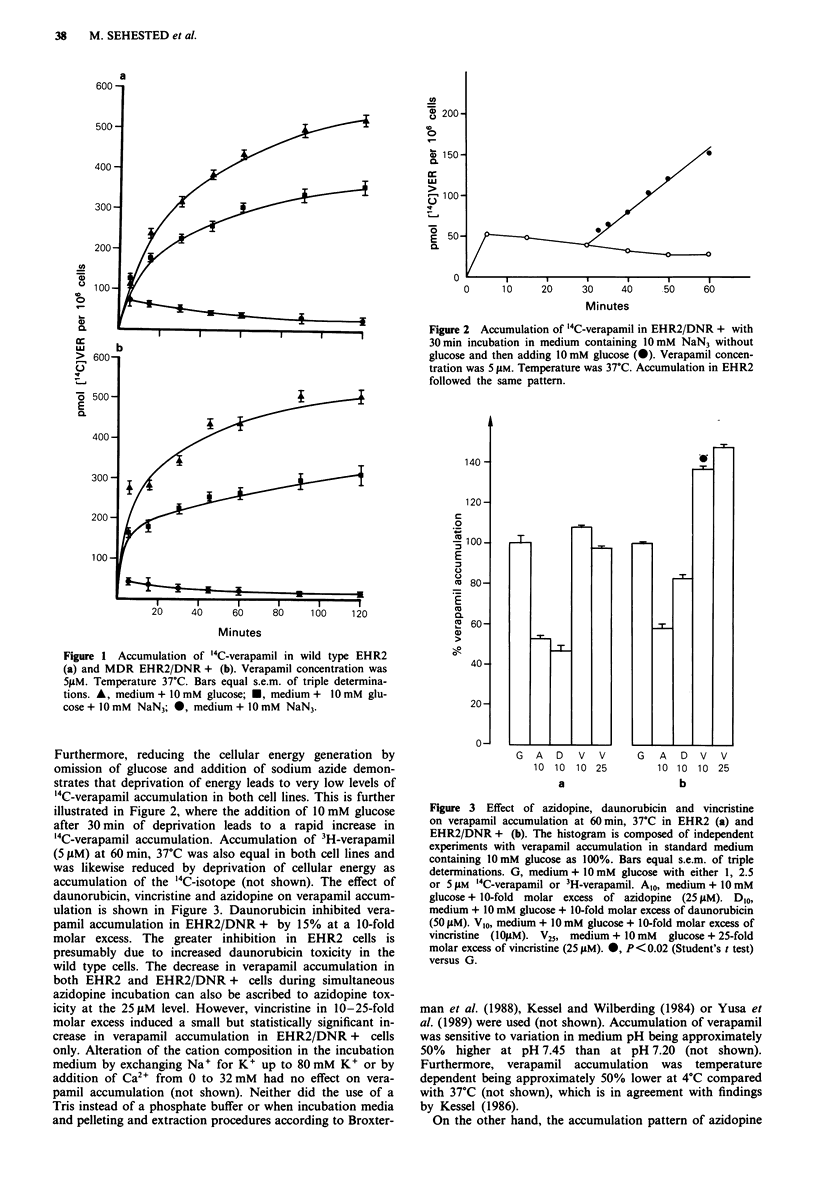

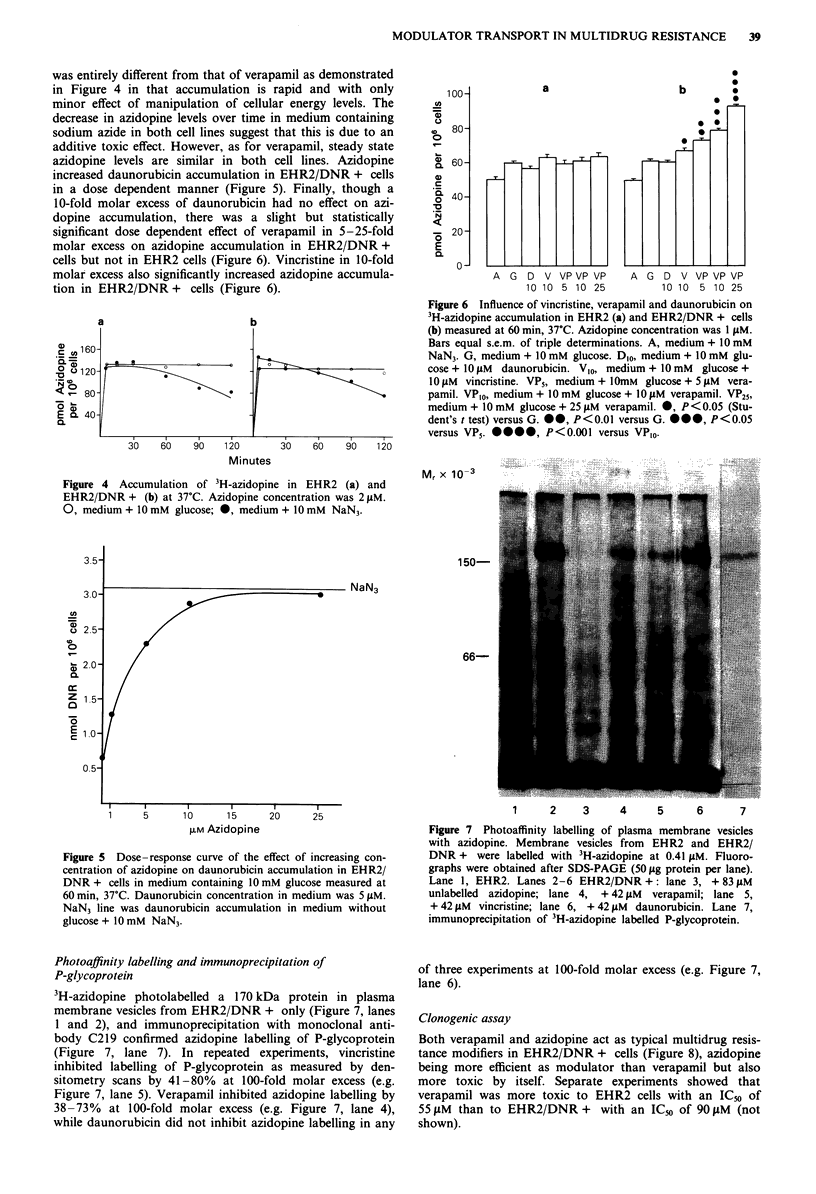

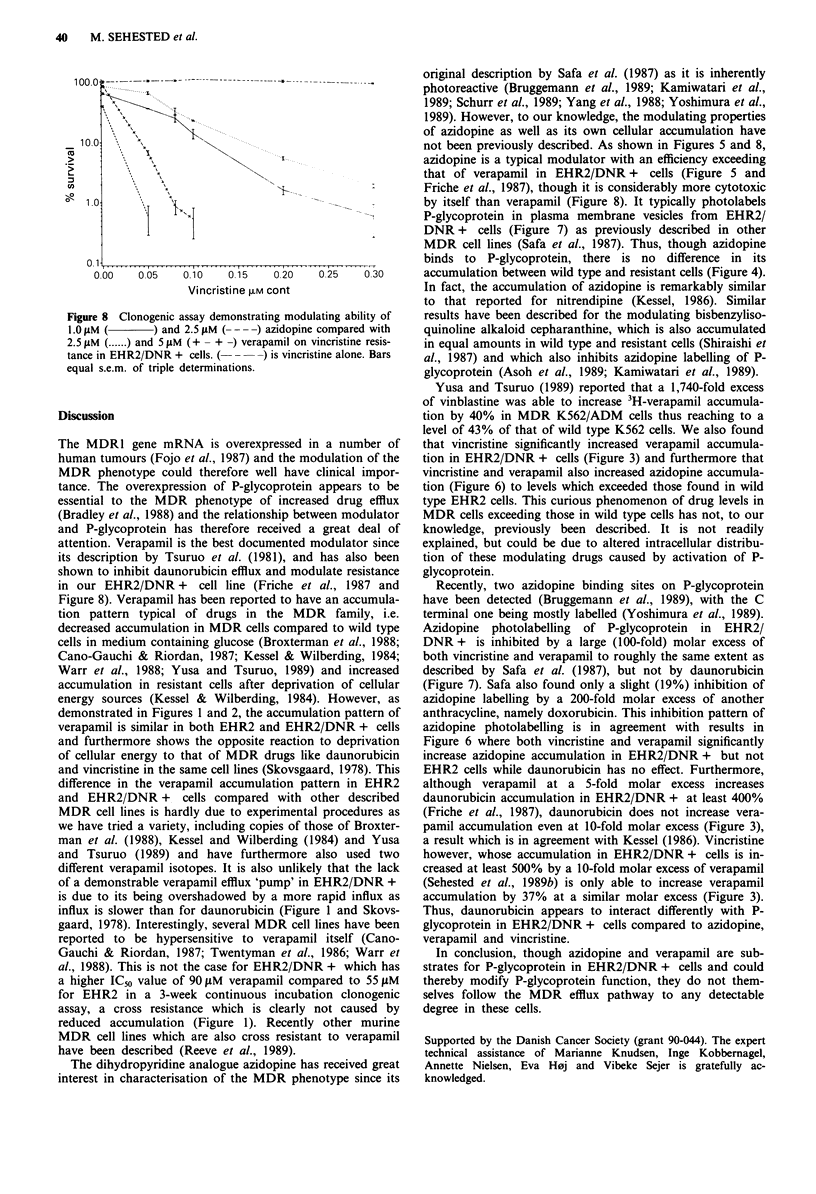

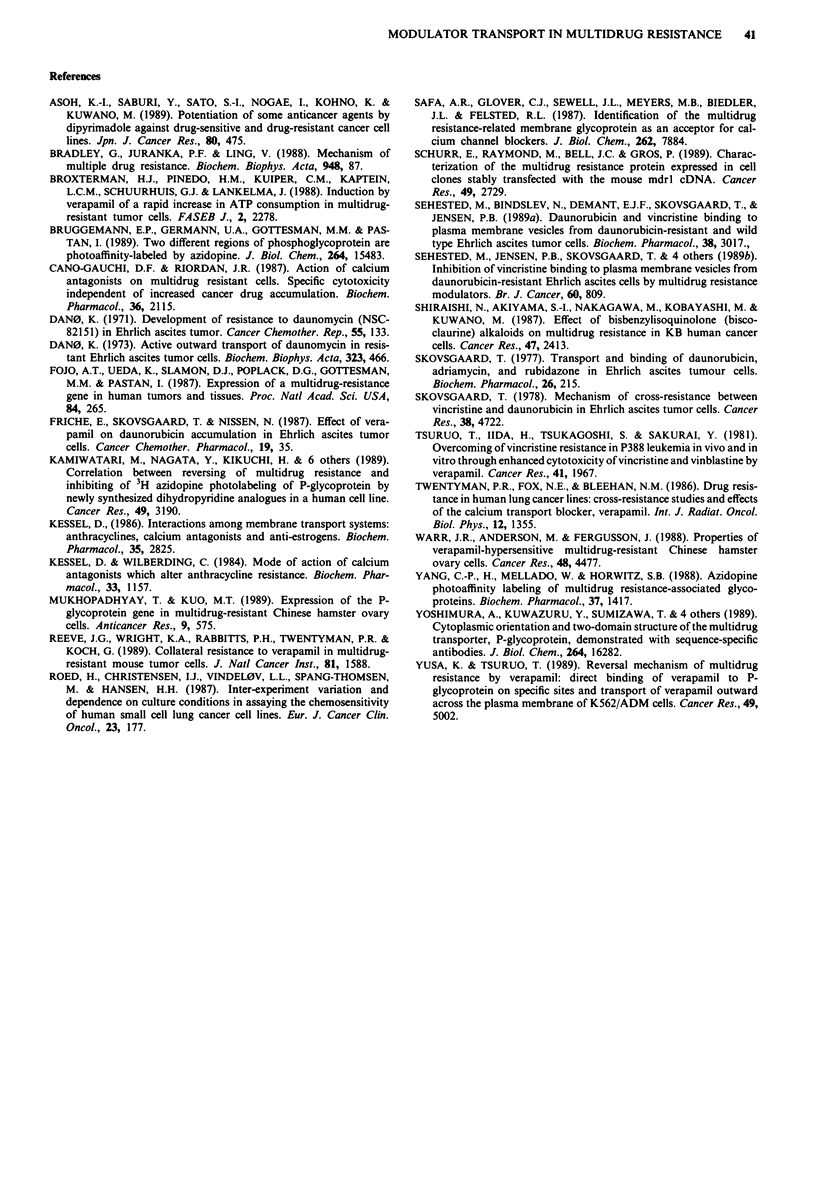


## References

[OCR_00578] Asoh K., Saburi Y., Sato S., Nogae I., Kohno K., Kuwano M. (1989). Potentiation of some anticancer agents by dipyridamole against drug-sensitive and drug-resistant cancer cell lines.. Jpn J Cancer Res.

[OCR_00584] Bradley G., Juranka P. F., Ling V. (1988). Mechanism of multidrug resistance.. Biochim Biophys Acta.

[OCR_00588] Broxterman H. J., Pinedo H. M., Kuiper C. M., Kaptein L. C., Schuurhuis G. J., Lankelma J. (1988). Induction by verapamil of a rapid increase in ATP consumption in multidrug-resistant tumor cells.. FASEB J.

[OCR_00596] Bruggemann E. P., Germann U. A., Gottesman M. M., Pastan I. (1989). Two different regions of P-glycoprotein [corrected] are photoaffinity-labeled by azidopine.. J Biol Chem.

[OCR_00599] Cano-Gauci D. F., Riordan J. R. (1987). Action of calcium antagonists on multidrug resistant cells. Specific cytotoxicity independent of increased cancer drug accumulation.. Biochem Pharmacol.

[OCR_00608] Dano K. (1973). Active outward transport of daunomycin in resistant Ehrlich ascites tumor cells.. Biochim Biophys Acta.

[OCR_00605] Dano K. (1971). Development of resistance to daunomycin (NSC-82151) in Ehrlich ascites tumor.. Cancer Chemother Rep.

[OCR_00611] Fojo A. T., Ueda K., Slamon D. J., Poplack D. G., Gottesman M. M., Pastan I. (1987). Expression of a multidrug-resistance gene in human tumors and tissues.. Proc Natl Acad Sci U S A.

[OCR_00617] Friche E., Skovsgaard T., Nissen N. I. (1987). Effect of verapamil on daunorubicin accumulation in Ehrlich ascites tumor cells.. Cancer Chemother Pharmacol.

[OCR_00622] Kamiwatari M., Nagata Y., Kikuchi H., Yoshimura A., Sumizawa T., Shudo N., Sakoda R., Seto K., Akiyama S. (1989). Correlation between reversing of multidrug resistance and inhibiting of [3H]azidopine photolabeling of P-glycoprotein by newly synthesized dihydropyridine analogues in a human cell line.. Cancer Res.

[OCR_00629] Kessel D. (1986). Interactions among membrane transport systems: anthracyclines, calcium antagonists and anti-estrogens.. Biochem Pharmacol.

[OCR_00634] Kessel D., Wilberding C. (1984). Mode of action of calcium antagonists which alter anthracycline resistance.. Biochem Pharmacol.

[OCR_00639] Mukhopadhyay T., Kuo M. T. (1989). Expression of the P-glycoprotein gene in multidrug-resistant Chinese hamster ovary cells.. Anticancer Res.

[OCR_00644] Reeve J. G., Wright K. A., Rabbitts P. H., Twentyman P. R., Koch G. (1989). Collateral resistance to verapamil in multidrug-resistant mouse tumor cells.. J Natl Cancer Inst.

[OCR_00649] Roed H., Christensen I. B., Vindeløv L. L., Spang-Thomsen M., Hansen H. H. (1987). Inter-experiment variation and dependence on culture conditions in assaying the chemosensitivity of human small cell lung cancer cell lines.. Eur J Cancer Clin Oncol.

[OCR_00656] Safa A. R., Glover C. J., Sewell J. L., Meyers M. B., Biedler J. L., Felsted R. L. (1987). Identification of the multidrug resistance-related membrane glycoprotein as an acceptor for calcium channel blockers.. J Biol Chem.

[OCR_00662] Schurr E., Raymond M., Bell J. C., Gros P. (1989). Characterization of the multidrug resistance protein expressed in cell clones stably transfected with the mouse mdr1 cDNA.. Cancer Res.

[OCR_00668] Sehested M., Bindslev N., Demant E. J., Skovsgaard T., Jensen P. B. (1989). Daunorubicin and vincristine binding to plasma membrane vesicles from daunorubicin-resistant and wild type Ehrlich ascites tumor cells.. Biochem Pharmacol.

[OCR_00674] Sehested M., Jensen P. B., Skovsgaard T., Bindslev N., Demant E. J., Friche E., Vindeløv L. (1989). Inhibition of vincristine binding to plasma membrane vesicles from daunorubicin-resistant Ehrlich ascites cells by multidrug resistance modulators.. Br J Cancer.

[OCR_00680] Shiraishi N., Akiyama S., Nakagawa M., Kobayashi M., Kuwano M. (1987). Effect of bisbenzylisoquinoline (biscoclaurine) alkaloids on multidrug resistance in KB human cancer cells.. Cancer Res.

[OCR_00691] Skovsgaard T. (1978). Mechanism of cross-resistance between vincristine and daunorubicin in Ehrlich ascites tumor cells.. Cancer Res.

[OCR_00686] Skovsgaard T. (1977). Transport and binding of daunorubicin, adriamycin, and rubidazone in Ehrlich ascites tumour cells.. Biochem Pharmacol.

[OCR_00696] Tsuruo T., Iida H., Tsukagoshi S., Sakurai Y. (1981). Overcoming of vincristine resistance in P388 leukemia in vivo and in vitro through enhanced cytotoxicity of vincristine and vinblastine by verapamil.. Cancer Res.

[OCR_00702] Twentyman P. R., Fox N. E., Bleehen N. M. (1986). Drug resistance in human lung cancer cell lines: cross-resistance studies and effects of the calcium transport blocker, verapamil.. Int J Radiat Oncol Biol Phys.

[OCR_00708] Warr J. R., Anderson M., Fergusson J. (1988). Properties of verapamil-hypersensitive multidrug-resistant Chinese hamster ovary cells.. Cancer Res.

[OCR_00713] Yang C. P., Mellado W., Horwitz S. B. (1988). Azidopine photoaffinity labeling of multidrug resistance-associated glycoproteins.. Biochem Pharmacol.

[OCR_00718] Yoshimura A., Kuwazuru Y., Sumizawa T., Ichikawa M., Ikeda S., Uda T., Akiyama S. (1989). Cytoplasmic orientation and two-domain structure of the multidrug transporter, P-glycoprotein, demonstrated with sequence-specific antibodies.. J Biol Chem.

[OCR_00724] Yusa K., Tsuruo T. (1989). Reversal mechanism of multidrug resistance by verapamil: direct binding of verapamil to P-glycoprotein on specific sites and transport of verapamil outward across the plasma membrane of K562/ADM cells.. Cancer Res.

